# Higher versus lower blood pressure targets after cardiac arrest: A meta-analysis of randomized controlled trials

**DOI:** 10.1016/j.ihj.2023.06.005

**Published:** 2023-06-14

**Authors:** Huzaifa Ahmad Cheema, Arman Shafiee, Mohammad Mobin Teymouri Athar, Amirhossein Akhondi, Abia Shahid, Muhammad Saqib Ghafoor, Farah Yasmin, Abdulqadir J. Nashwan, Anoop Titus

**Affiliations:** a Department of Cardiology, King Edward Medical University, Lahore, Pakistan; b Clinical Research Development Unit, Alborz University of Medical Sciences, Karaj, Iran; c Student Research Committee, School of Medicine, Alborz University of Medical Sciences, Karaj, Iran; d School of Medicine, Shahid Beheshti University of Medical Sciences, Tehran, Iran; e Department of Medicine, King Edward Medical University, Lahore, Pakistan; f Department of Internal Medicine, Dow Medical College, Dow University of Health Sciences, Karachi, Pakistan; g Hamad Medical Corporation, Doha, Qatar; h Department of Internal Medicine, Saint Vincent Hospital, Worcester, MA, USA

**Keywords:** Blood pressure, Mean arterial pressure, Cardiac arrest, Meta-analysis

## Abstract

A few mostly underpowered randomized controlled trials (RCTs) have been used to study the impact of blood pressure (BP) targets in out-of-hospital cardiac arrest (OHCA) patients. We aimed to perform an updated meta-analysis to compare the outcomes between the higher BP target and the lower BP target groups following OHCA. A systematic search was conducted on PubMed, Embase and the Cochrane Library until December 2022. We pooled odds ratios (ORs) and mean differences (MDs) with 95% conﬁdence intervals (CIs) using RevMan 5.4. Our search yielded four RCTs with a total of 1114 patients. Regarding our primary outcome of all-cause mortality, there was no significant difference between higher versus lower BP target goals in post-OHCA patients (OR 1.12, 95% CI: 0.86 to 1.45). Furthermore, there were no significant differences between the two groups in good neurological outcome, the incidence of arrhythmia, need for renal replacement therapy, and the levels of neuron-specific enolase at 48 h. The length of ICU stay of patients treated with the higher BP target was significantly lower but by a small margin. These findings do not support the use of a higher BP target but are subject to confirmation by large-scale RCTs investigating homogenous BP goals.

## 1 Introduction

Out-of-hospital cardiac arrest (OHCA) is a threatening condition with an estimated mortality rate of 30–50%.^1^ Unstable hemodynamics and systemic ischemia/reperfusion after cardiac arrest result in hypoperfusion of the brain leading to hypoxic-ischemic brain injury.^2^

Historically, maintaining optimal temperature has been the focus for managing OHCA patients.^3^ Blood pressure (BP) monitoring and targeting higher BP has been suggested as a hypothetical treatment for OHCA patients.^4^ There is evidence that shows mortality and poor neurological outcomes are significantly related to the hypotension state of these patients after cardiac arrest.^5^ However, most of the data available which report favorable outcomes for the higher BP target group compared with the lower BP target group are observational in nature and evidence for a specific high BP target is limited. Currently, the guidelines of the European Resuscitation Council and European Society of Intensive Care Medicine recommend avoiding hypotension by maintaining a mean arterial pressure (MAP) greater than 65 mm Hg for post-cardiac arrest patients.^6^ Due to the paucity of data, there is a need to establish a specific BP target for post-OHCA patients using robust data that ameliorates clinical outcomes in these patients.

Only a few mostly underpowered randomized controlled trials (RCTs) have been used to study the impact of BP targets on post-resuscitation care in OHCA patients. The latest study by Møller et al is the largest double-blind RCT to date that allows us to take a deeper dive into understanding the outcomes of the use of goal-directed MAP in OHCA.^7^ We aimed to perform a meta-analysis of the results of the available evidence to compare the outcomes in the higher BP target group versus the lower BP target group following OHCA.

## 2 Methods

Our pre-registered meta-analysis (PROSPERO CRD42022374616) was conducted in accordance with the Cochrane Handbook for Systematic Reviews of Intervention. A systematic search was conducted on MEDLINE (via PubMed), Embase and the Cochrane Library until December 2022 to find relevant articles. Additionally, the reference lists of related articles were hand-searched. Each article was evaluated based on its title/abstract, and full text by two individual reviewers. The inclusion criteria for our meta-analysis based on the PICOS definition were: (1) population: patients with out-of-hospital cardiac arrest hospitalized in the intensive care unit; (2) intervention: a high BP goal; (3) control: a low BP goal; (4) outcome: reporting relevant outcomes mentioned below; (5) study type: RCTs only. We excluded non-randomized trials, observational studies, and reviews. Our primary endpoint was all-cause mortality at 90 days (if mortality at 90 days was not reported the timepoint closest to it was used) while the secondary outcomes were: (1) level of neuron-specific enolase at 48 h; (2) length of intensive care unit (ICU) stay; (3) incidence of arrhythmia; (4) need for renal replacement therapy; and (5) proportion of patients with good neurological outcome (Cerebral Performance Categories [CPC] Scale 1–2).

The quality of each trial was assessed using the Cochrane Risk of Bias 2 (RoB 2.0) tool. A random-effects meta-analysis was performed. We used odds ratios (ORs) for dichotomous outcomes and mean differences (MDs) for continuous outcomes with corresponding 95% conﬁdence intervals (CIs). I^2^ value was used to report heterogeneity among included studies. All statistical analyses were done in RevMan version 5.4.

## 3 Results

Our search yielded four RCTs with a total of 1114 patients.^7^, ^8^, ^9^, ^10^ The detailed characteristics of each trial are presented in Table 1. One study was found to have some concerns of bias, due to issues in the domain of deviations from intended interventions,^9^ while the rest of the studies were judged to be at low risk of bias (Table 1).

**Table 1:** Characteristics of the included trials.

ID	Author	Year	Country	Population	Total number of patients	Age	Follow up	MAP target (mm Hg)	Risk of bias
1	Ameloot, K.	2019	Belgium	Out-of-hospital cardiac arrest	112	NA	180 days	85-100 (early goal-directed hemodynamic optimization strategy= (EGDHO)) vs 65	Low
2	Grand, J.	2020	Denmark	Adult (≥18 years), comatose (Glasgow Coma Score ≤8) resuscitated out-of-hospital cardiac arrest patients	77	MAP 65: 59 (±13) MAP 72: 63 (±10)	180 days	72 vs 65	Low
3	Pekka Jakkula	2018	Finland	Out-of-hospital cardiac arrest	123	Low-normal MAP group: 61 ± 11 High-normal MAP group: 58 ± 14	180 days	80-100 vs. 65-75	Some concerns
4	Kjaergaard, J.	2022	Denmark	Comatose adults who had been resuscitated after an out-of-hospital cardiac arrest	802	High blood pressure: 63 ± 13 Low Blood-Pressure: 62 ± 14	90 days	77 vs. 63	Low

MAP, mean arterial pressure; NA, not applicable.

**Fig. 1:**
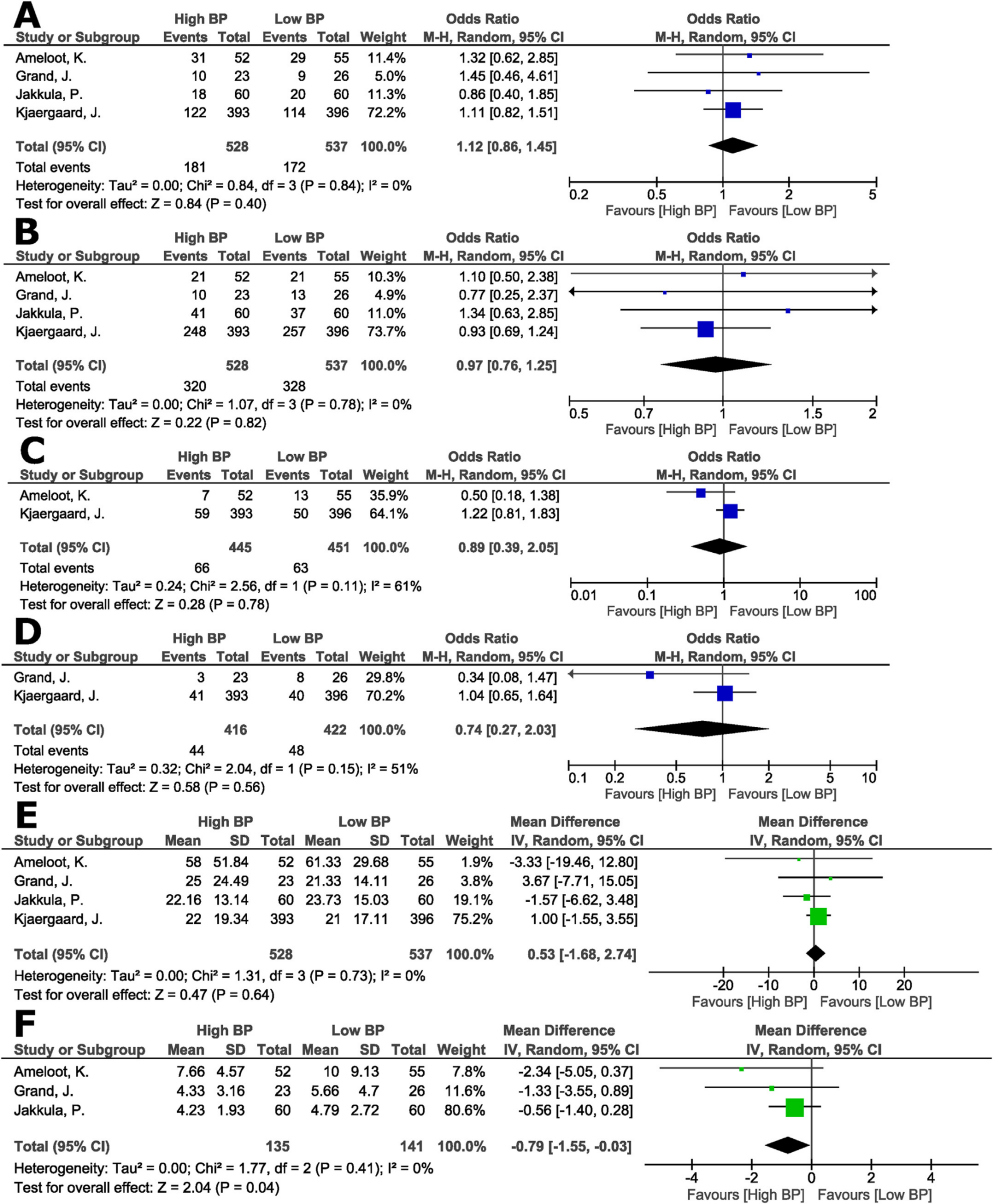
Effect of higher blood pressure (BP) targets versus lower BP targets on: A) all-cause mortality; B) good neurological outcome; C) the incidence of arrhythmia; D) need for renal replacement therapy; E) levels of neuron-specific enolase at 48 h; and F) length of ICU stay.

The result regarding the primary endpoint of mortality showed no significant difference between higher versus lower BP target goals in post-OHCA patients (OR 1.12, 95% CI: 0.86 to 1.45; I^2^ = 0%; Fig. 1A). Furthermore, there were no significant differences between the two groups in good neurological outcome (OR 0.97, 95% CI: 0.76 to 1.25, I^2^ = 0%; Fig. 1B), the incidence of arrhythmia (OR 0.89, 95% CI: 0.39 to 2.05, I^2^ = 61%; Fig. 1C), need for renal replacement therapy (OR 0.74, 95% CI: 0.27 to 2.03, I^2^ = 51%; Fig. 1D) and the levels of neuron-specific enolase at 48 h (MD 0.53, 95% CI: −0.09 to 0.15, I^2^ = 0%; Fig. 1E). The length of ICU stay of patients treated with the higher BP target was significantly lower (MD -0.79 days, 95% CI: −1.55 to −0.03, I^2^ = 0%; Fig. 1F).

## 4 Discussion

To our knowledge, this is the first meta-analysis conducted on the latest evidence to assess BP targets in patients after OHCA in the critical care setting. Notably, we found that there was no significant difference in all-cause mortality between higher BP and lower BP target groups. However, it is noteworthy to mention that the length of ICU stay in the high BP goal group was lower, albeit by a small margin, which is contrary to the individual result of each included trial.

Similar to the results of our study, a recent meta-analysis done in critical patients by Sarkar et al. demonstrated no significant difference in mortality, and duration of mechanical ventilation between the two groups^11^; however, the included population contained mostly patients with septic shock rather than OHCA. Contrary to their results which showed no reduction in the duration of ICU stay, we found a shorter ICU length of stay in the higher BP target group.

Our study has some limitations. The limited number of RCTs available means that our meta-analysis might be underpowered to detect a benefit if it exists. Furthermore, although there was generally low heterogeneity in our outcomes, the BP targets for the intervention and control groups varied across the RCTs. Future RCTs should investigate similar BP targets in order to determine the optimal target.

In conclusion, treatment with a higher BP target in OHCA patients reduced the length of ICU stay by a small margin but did not improve mortality or any other reported clinical outcome. These findings do not support the use of a higher BP target in these patients but are subject to confirmation by future large-scale RCTs investigating homogenous BP goals.

## Statements and declarations

### Financial support

No financial support was received for this study.

## Declaration of competing interest

The authors declare that they have no known competing financial interests or personal relationships that could have appeared to influence the work reported in this paper.
